# Supplementary figures and images for: Adipose tissue–specific ablation of Ces1d causes metabolic dysregulation in mice
Source: Life Sci Alliance. 2022 Apr 22;5(8):e202101209. doi: 10.26508/lsa.202101209 (PMC9034061; doi:10.26508/lsa.202101209)

Raw images of immunoblots in Fig 3B

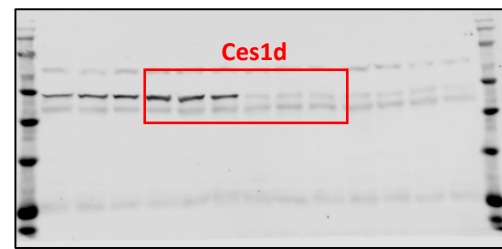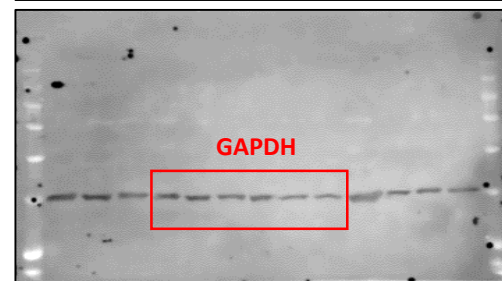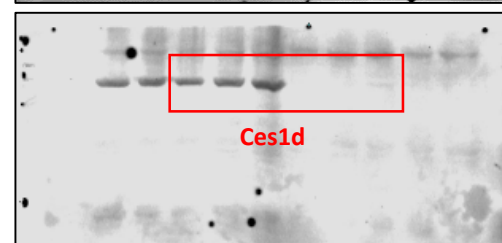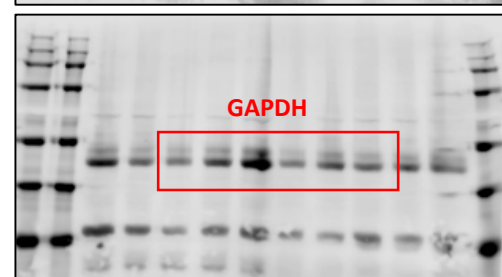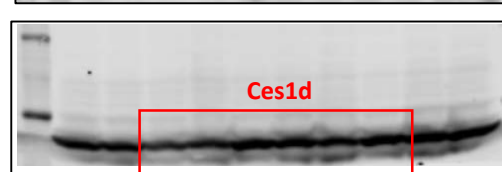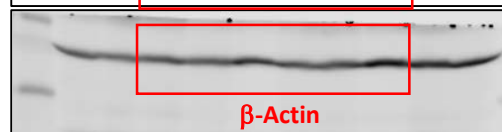

Supplement: Supplementary file 2 [file LSA-2021-01209_SdataF3.pdf]

Raw images of immunoblots in Fig 4K

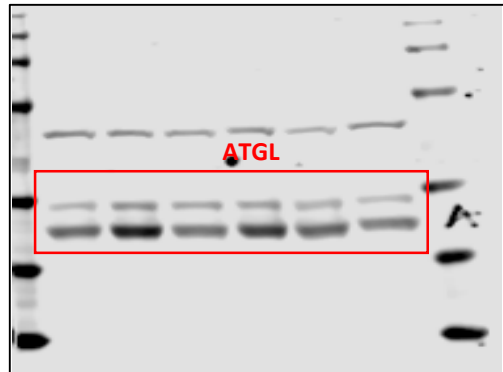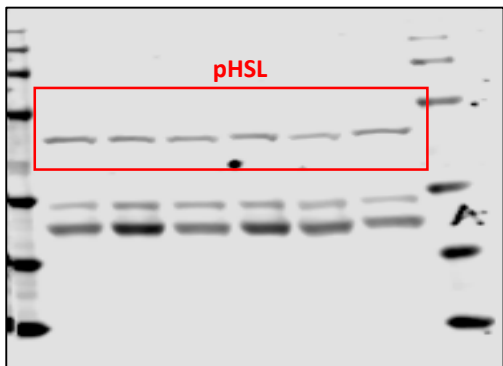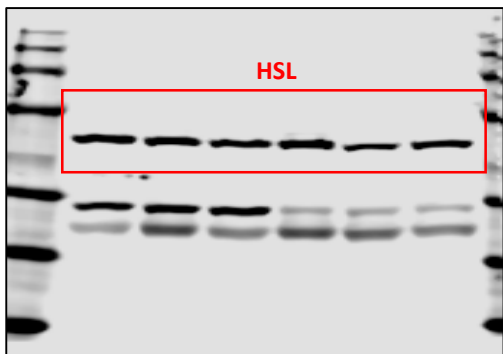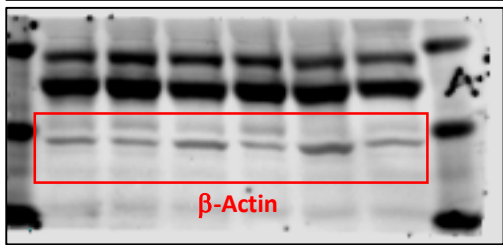

Raw images of immunoblots in Fig 4L

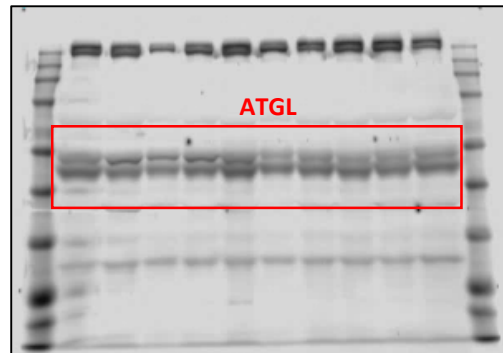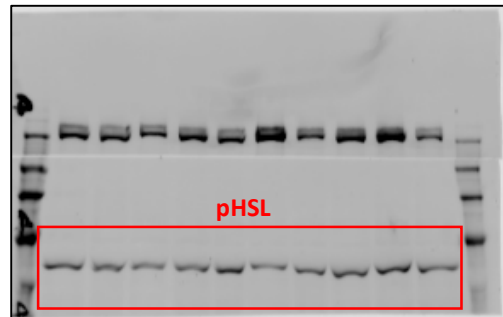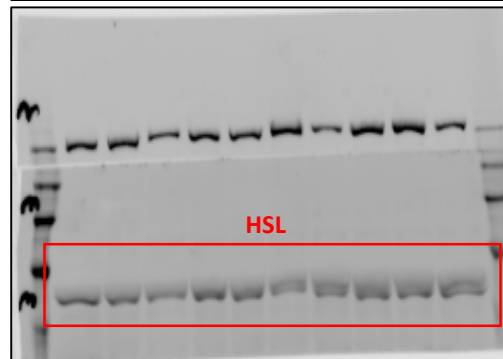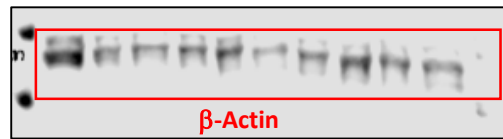

Supplement: Supplementary file 3 [file LSA-2021-01209_SdataF4.pdf]

Raw images of immunoblots in Fig 7M

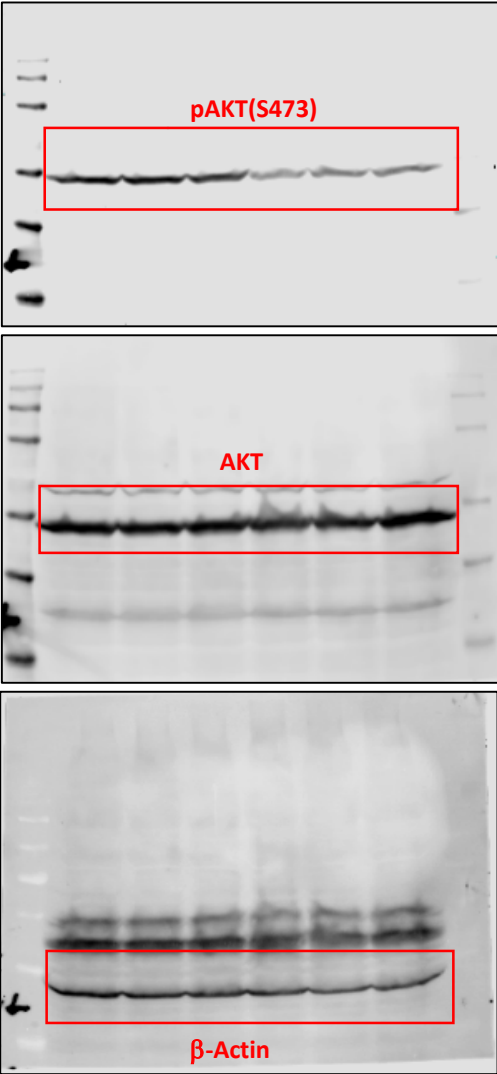

Raw images of immunoblots in Fig 7O

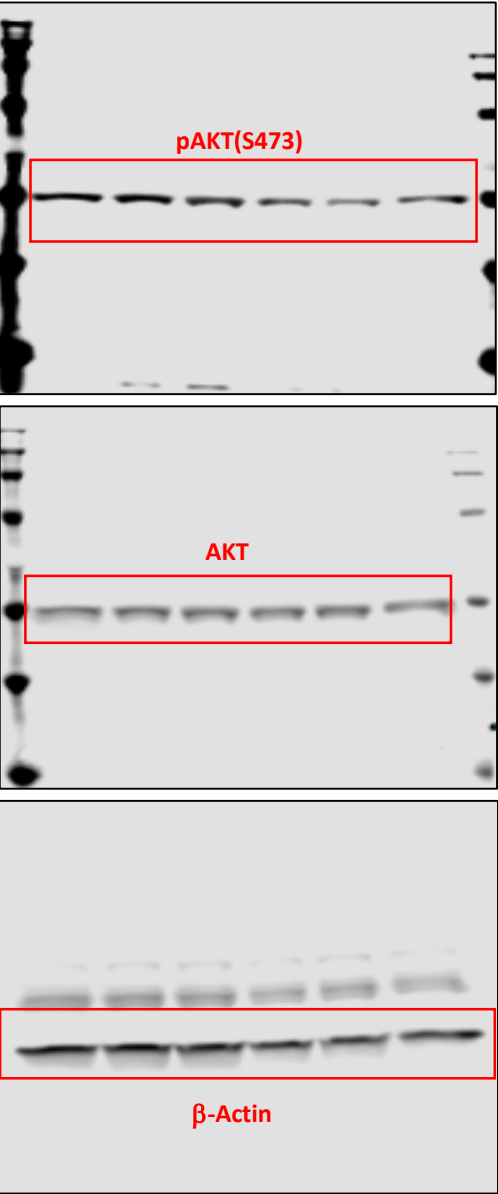

Supplement: Supplementary file 6 [file LSA-2021-01209_SdataF7.pdf]
