# Supplementary material for: Adipose tissue–specific ablation of Ces1d causes metabolic dysregulation in mice
Source: Life Sci Alliance. 2022 Apr 22;5(8):e202101209. doi: 10.26508/lsa.202101209 (PMC9034061; doi:10.26508/lsa.202101209)

Raw images of immunoblots in FigS2A

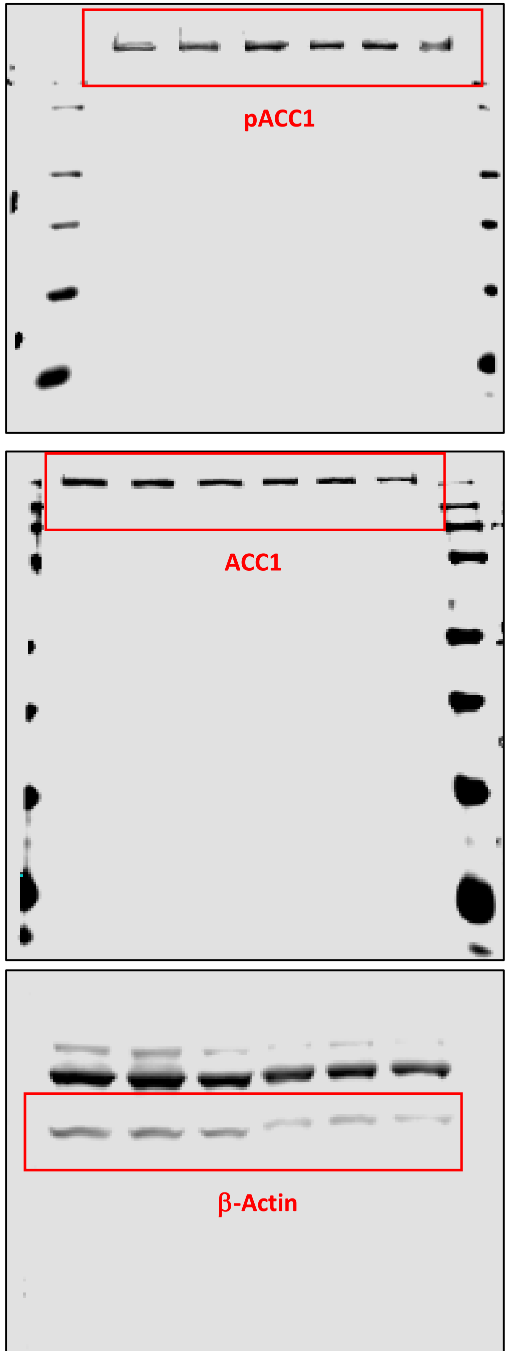

Raw images of immunoblots in FigS2C

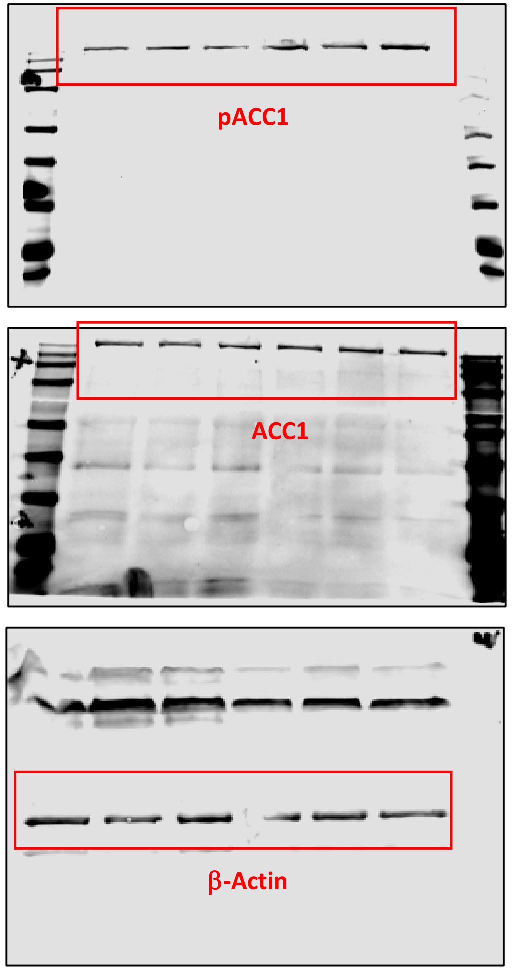

Raw images of immunoblots in FigS2E

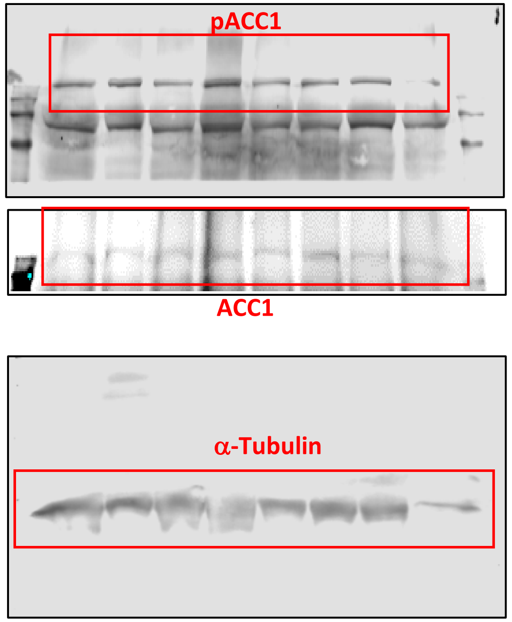

Raw images of immunoblots in FigS2G

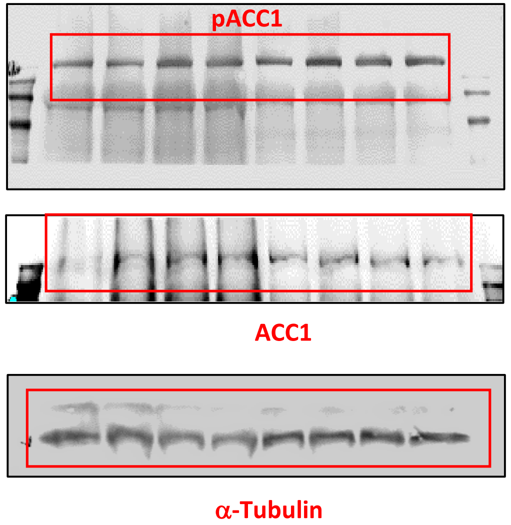

Supplement: Supplementary file 5 [file LSA-2021-01209_SdataFS2.pdf]
