# Supplementary material for: Adipose tissue–specific ablation of Ces1d causes metabolic dysregulation in mice
Source: Life Sci Alliance. 2022 Apr 22;5(8):e202101209. doi: 10.26508/lsa.202101209 (PMC9034061; doi:10.26508/lsa.202101209)
Supplement: Supplementary file 7 [file LSA-2021-01209_TableS1.docx]

Table S1. Full names of the lipid species in lipidomic analysis

**Abbreviation** **Full name**

Cer Ceramides

dhCer Dihydroceramides

CL Cardiolipin

DG diglyceride

PA phosphatidic acid

PC phosphatidylcholine

PE phosphatidylethanolamine

PG phosphatidylglycerol

PI phosphatidylinositol

PIP phosphatidylinositol-P

PS phosphatidylserine

SM sphingomyelin
