# Supplementary material for: Adipose tissue–specific ablation of Ces1d causes metabolic dysregulation in mice
Source: Life Sci Alliance. 2022 Apr 22;5(8):e202101209. doi: 10.26508/lsa.202101209 (PMC9034061; doi:10.26508/lsa.202101209)

Raw images of immunoblots in Fig 8B

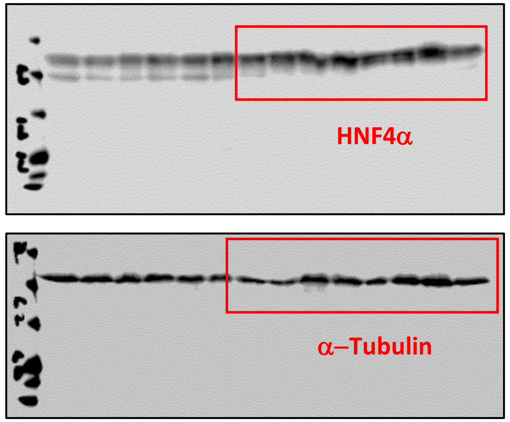

Raw images of immunoblots in Fig 8G

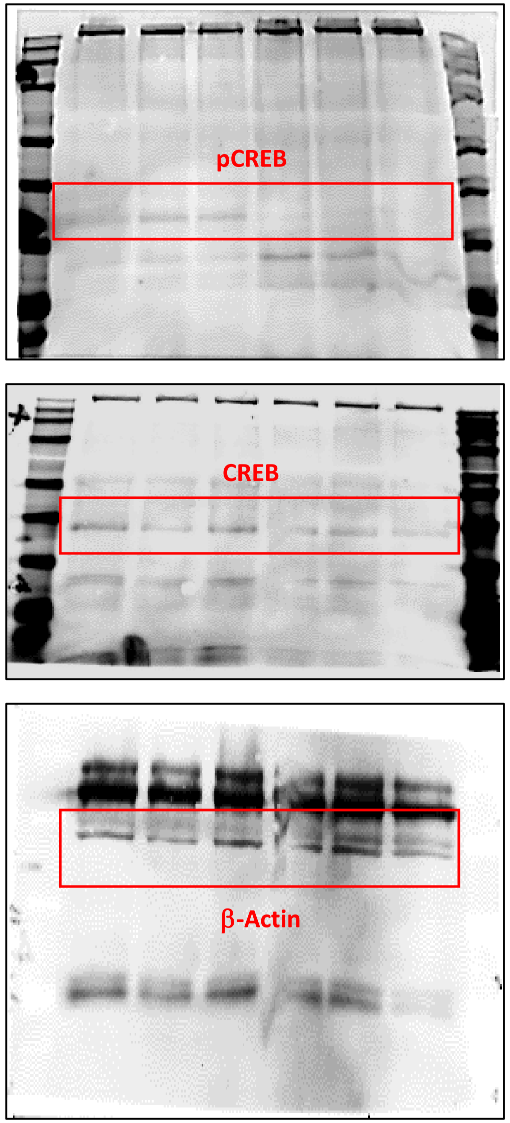

Raw images of immunoblots in Fig 8I

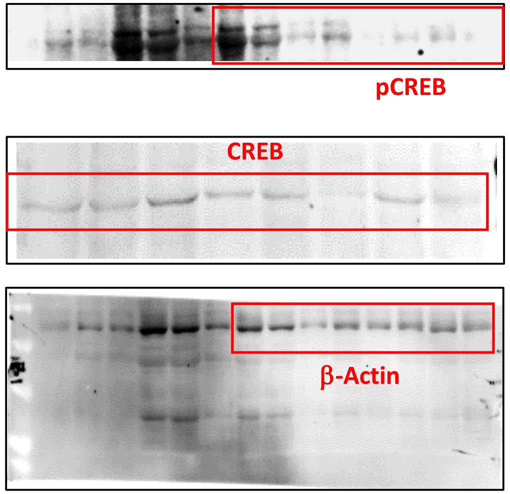

Raw images of immunoblots in Fig 8K

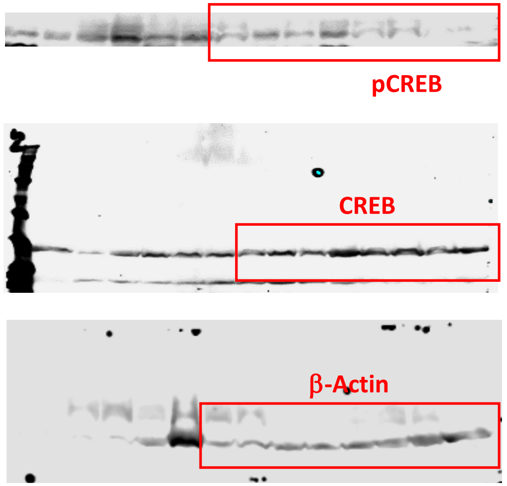

Supplement: Supplementary file 8 [file LSA-2021-01209_SdataF8.pdf]
