# Supplementary material for: Adipose tissue–specific ablation of Ces1d causes metabolic dysregulation in mice
Source: Life Sci Alliance. 2022 Apr 22;5(8):e202101209. doi: 10.26508/lsa.202101209 (PMC9034061; doi:10.26508/lsa.202101209)
Supplement: Supplementary file 9 [file LSA-2021-01209_TableS2.docx]

Table S2. The down-regulated pathways in WAT of the Ces1d FKO mice

| Pathway name | P value | False discovery rate (FDR) | Differentially expressed genes |
| --- | --- | --- | --- |
| Ribosome | 2.33E-15 | 1.82E-12 | *Rpl35;Rpl23a;Rpl5;Mrps27;Rpl30;Mrpl42;Rpl11;Rpl31;Mrps21;Rpl19;Rpl22;Rpl28;2810006K23Rik;Rps15;Rps3a1;Rpl41;Rpl7a;Rpl36a;Rpsa;Mrpl58;Rpl13a;Rps27rt;Rpl14;Rps21;Mpv17l2;Mrpl34;Mrpl14;Mrps33;Mrps15;Rpl18a;Mrps35;Rpl32* |
| Parkinson disease | 4.55E-15 | 2.37E-12 | *Uqcrh;Atp5h;Ndufa3;Cox5b;Park7;Uqcr11;Uqcrb;Cox8a;Ndufv3;Ndufb8;Cox6c;Ndufs5;Ndufa1;Atp5g2;Cox7b;Cox7a2l;Ndufa10;Ndufb11;Gnai1;Ndufb10;Ndufb2;Cox6b1;Cox4i1;Ndufs6;Ndufb7* |
| Oxidative phosphorylation | 1.09E-14 | 3.47E-12 | *Uqcrh;Atp5h;Atp5l;Ndufa3;Cox5b;Uqcr11;Uqcrb;Cox8a;Ndufv3;Ndufb8;Cox6c;Ndufs5;Ndufa1;Atp5g2;Cox7b;Cox7a2l;Ndufa10;Ndufb11;Ndufb10;Ndufb2;Cox6b1;Cox4i1;Ndufs6;Ndufb7* |
| Mitochondrial protein complex | 1.11E-14 | 3.47E-12 | *Mrps27;Uqcrh;Mrpl42;Atp5h;Mrps21;Atp5l;Pdk1;Ndufa3;Timm17b;Park7;2810006K23Rik;Ndufv3;Mrpl58;Bckdk;Ndufb8;Ndufs5;Ndufa1;Atp5g2;Usmg5;Mpv17l2;Cox7a2l;Mrpl34;Mrpl14;Mrps33;Ndufa10;Ndufb11;Mrps15;Ndufb10;Ndufb2;Cox4i1;Ndufs6;Mrps35;Ndufb7* |
| Structural constituent of ribosome | 4.24E-14 | 1.10E-11 | *Rpl35;Rpl23a;Rpl5;Rpl30;Rpl11;Rpl31;Mrps21;Rpl19;Rpl22;Rpl28;Rps15;Rps3a1;Rpl41;Rpl36a;Rpsa;Rpl13a;Rps27rt;Rpl14;Rps21;Mrpl34;Mrpl14;Mrps15;Rpl18a;Mrps35;Rpl32* |
| Respiratory chain | 7.53E-14 | 1.68E-11 | *Uqcrh;Ndufa3;Park7;Uqcr11;Cox8a;Ndufv3;Ndufb8;Ndufs5;Ndufa1;Cox7b;Cox7a2l;Ndufa10;Ndufb11;Ndufb10;Ndufb2;Cox6b1;Cox4i1;Ndufs6;Ndufb7* |
| Organelle inner membrane | 1.89E-12 | 3.29E-10 | *Uqcrh;Emd;Aifm1;Atp5h;Dnajc30;Mrps21;Atp5l;Tmem11;Pet100;Ndufa3;Timm17b;Park7;Uqcr11;Cox8a;Slc25a23;Ndufv3;Ndufb8;Cox6c;Ndufs5;Ndufa1;Mpc1;Oxa1l;Atp5g2;Maob;Usmg5;Cox7b;Mpv17l2;Cox7a2l;Coa3;Ndufa10;Ndufb11;Chdh;Ndufb10;Ndufb2;Dhodh;Cox6b1;Cox4i1;Ndufs6;Ndufb7;Nipsnap1;Sfxn1;Tmem14c* |
| Alzheimer disease | 1.90E-12 | 3.29E-10 | *Uqcrh;Atp5h;Ndufa3;Cox5b;Uqcr11;Uqcrb;Cox8a;Ndufv3;Ndufb8;Cox6c;Ndufs5;Ndufa1;Hsd17b10;Atp5g2;Apoe;Cox7b;Cox7a2l;Ndufa10;Ndufb11;Ndufb10;Ndufb2;Cox6b1;Cox4i1;Ndufs6;Ndufb7* |
| Huntington disease | 2.69E-11 | 4.20E-09 | *Polr2j;Uqcrh;Sod1;Atp5h;Ndufa3;Cox5b;Uqcr11;Uqcrb;Cox8a;Ndufv3;Ndufb8;Cox6c;Ndufs5;Ndufa1;Atp5g2;Cox7b;Cox7a2l;Ndufa10;Ndufb11;Ndufb10;Ndufb2;Cox6b1;Cox4i1;Ndufs6;Ndufb7* |
| mitochondrial membrane part | 4.81E-11 | 6.54E-09 | *Uqcrh;Atp5h;Atp5l;Tmem11;Pet100;Ndufa3;Timm17b;Park7;Ndufv3;Ndufb8;Ndufs5;Ndufa1;Mpc1;Oxa1l;Atp5g2;Usmg5;Cox7b;Cox7a2l;Coa3;Ndufa10;Ndufb11;Ndufb10;Ndufb2;Cox4i1;Ndufs6;Ndufb7* |
| Non-alcoholic fatty liver disease (NAFLD) | 5.03E-11 | 6.54E-09 | *Adipor1;Uqcrh;Ndufa3;Cox5b;Uqcr11;Uqcrb;Cox8a;Ndufv3;Ndufb8;Cox6c;Ndufs5;Ndufa1;Cox7b;Cox7a2l;Ndufa10;Ndufb11;Ndufb10;Ndufb2;Cox6b1;Cox4i1;Ndufs6;Ndufb7* |
| Thermogenesis | 1.44E-10 | 1.72E-08 | *Uqcrh;Atp5h;Rps6;Atp5l;Ndufa3;Cox5b;Uqcr11;Uqcrb;Cox8a;Ndufv3;Ndufb8;Cox6c;Ndufs5;Ndufa1;Atp5g2;Cox7b;Cox7a2l;Coa3;Ndufa10;Ndufb11;Ndufb10;Ndufb2;Cox6b1;Cox4i1;Ndufs6;Ndufb7* |
| NADH dehydrogenase complex | 3.11E-10 | 3.47E-08 | *Ndufa3;Park7;Ndufv3;Ndufb8;Ndufs5;Ndufa1;Ndufa10;Ndufb11;Ndufb10;Ndufb2;Ndufs6;Ndufb7* |
| Oxidoreductase complex | 3.06E-09 | 3.19E-07 | *Uqcrh;Pdk1;Ndufa3;Park7;Ndufv3;Bckdk;Ndufb8;Ndufs5;Ndufa1;Pdha1;Ndufa10;Ndufb11;Ndufb10;Ndufb2;Ndufs6;Ndufb7* |
| NADH dehydrogenase complex assembly | 4.89E-09 | 4.77E-07 | *Aifm1;Ndufa3;Ndufb8;Ndufs5;Ndufa1;Oxa1l;Ndufa10;Ndufb11;Ndufb10;Ndufb2;Ndufb7* |
| Cytosolic part | 7.19E-08 | 6.60E-06 | *Rpl35;Rpl23a;Rpl5;Rpl30;Rpl11;Rpl31;Rpl19;Rpl22;Rpl28;Rps15;Rps3a1;Rpl41;Rpl7a;Rpl36a;Rpsa;Rpl13a;Rps27rt;Rpl14;Rps21;Ciao1;Prkca;Rpl18a;Rpl32* |
| Cardiac muscle contraction | 3.33E-07 | 2.89E-05 | *Uqcrh;Tpm1;Cox5b;Uqcr11;Uqcrb;Cox8a;Cox6c;Cox7b;Cacna2d1;Cox7a2l;Cox6b1;Cox4i1* |
| Generation of precursor metabolites and energy | 3.94E-06 | 0.000323905 | *Mecp2;Uqcrh;Aifm1;Epm2a;Dnajc30;Park7;Uqcr11;Cox8a;Slc25a23;Ndufv3;Ptges2;Phkb;Ndufb8;Cox6c;Oxa1l;Cox7b;Adh1;Cox7a2l;Pdha1;D2hgdh;Ndufa10;Stbd1;Crot;Por;Nr1d1;Cox4i1;Ndufs6* |
| Electron transfer activity | 1.01E-05 | 0.000789436 | *Uqcrh;Aifm1;Uqcr11;Cox8a;Ptges2;Cox6c;Cox7b;Cox7a2l;Por;Cox4i1* |
| Polysome | 1.74E-05 | 0.001295278 | *Epm2a;Rpl30;Rpl11;Rpl31;Rpl19;Rpl41;Rpl7a;Rps21;Rpl18a;Rpl32* |
| Oxidoreductase activity, acting on a heme group of donors | 2.12E-05 | 0.001504643 | *Cox8a;Cox6c;Cox7b;Cox7a2l;Por;Cox4i1* |
| Retrograde endocannabinoid signaling | 5.03E-05 | 0.003416813 | *Ndufa3;Ndufv3;Ndufb8;Ndufs5;Ndufa1;Ndufa10;Ndufb11;Prkca;Gnai1;Ndufb10;Ndufb2;Ndufs6;Ndufb7* |
| Mitochondrial matrix | 6.04E-05 | 0.00392903 | *Mrps27;Mrpl42;Mrps21;Clpp;Pdk1;Rida;Park7;2810006K23Rik;Mrpl58;Bckdk;Tfb2m;Mpv17l2;Pdha1;Mrpl34;Mrpl14;Mrps33;Ndufa10;Pccb;Mrps15;Mccc2;Aldh2;Mrps35* |
| Actin filament-based movement | 9.22E-05 | 0.005754717 | *Tpm1;Cacna1g;Myl6;Fxyd1;Myh14;Emp2;Scn3b;Pard3;Cacna2d1;Pdpn;Myo1b* |
| oxidoreductase activity, acting on NAD(P)H | 0.000133965 | 0.008043074 | *Cbr1;Aifm1;Nos3;Cyp2j6;Dhrs4;Ndufb8;Ndufa10;Por;Ndufb7* |
| Heme-copper terminal oxidase activity | 0.000215414 | 0.012454141 | *Cox8a;Cox6c;Cox7b;Cox7a2l;Cox4i1* |
| Cytoplasmic translation | 0.00044286 | 0.024689472 | *Rpl30;Rpl11;Rpl31;Rpl19;Rpl41;Rpl13a;Rps21;Rpl18a;Rpl32* |
| Ribose phosphate metabolic process | 0.000482133 | 0.025952072 | *Mecp2;Ces1d;Uqcrh;Atp5h;Dnajc30;Mcee;Atp5l;Pdk1;Park7;Slc25a23;Ndufv3;Ndufb8;Mpc1;Atp5g2;Cox7a2l;Papss1;Pdha1;Ndufa10;Mccc2;Dhodh;Crot;Cox4i1;Ndufs6;Ola1* |
| Cytochrome complex | 0.000746285 | 0.03883168 | *Uqcrh;Cox8a;Cox7b;Cox6b1;Cox4i1* |
| Sulfur compound metabolic process | 0.000941549 | 0.045929959 | *Ces1d;Spock3;Chst4;Sod1;Mcee;Pdk1;Park7;Cdo1;Mpc1;Gstp1;Papss1;Pdha1;Ciao1;Gstm4;Bola2;Ptges;Adi1* |
| Nucleoside monophosphate metabolic process | 0.000941549 | 0.045929959 | *Mecp2;Uqcrh;Atp5h;Dnajc30;Atp5l;Park7;Slc25a23;Dctd;Ndufv3;Ndufb8;Atp5g2;Cox7a2l;Ndufa10;Dhodh;Cox4i1;Ndufs6;Ola1* |
