# Supplementary material for: Adipose tissue–specific ablation of Ces1d causes metabolic dysregulation in mice
Source: Life Sci Alliance. 2022 Apr 22;5(8):e202101209. doi: 10.26508/lsa.202101209 (PMC9034061; doi:10.26508/lsa.202101209)
Supplement: Supplementary file 10 [file LSA-2021-01209_TableS3.docx]

Table S3: qPCR Primers

| Gene Name | Species | Sense Sequence (5' to 3') | Antisense Sequence (5' to 3) | Reference |
| --- | --- | --- | --- | --- |
| Creb1 | Mouse | CCAGTCTCCACAAGTCCAAACAG | GGCACTGTTACAGTGGTGATGG | 12 |
| Ces1d |  | GGAGAGTCAGCAGGAGGTTTC | GAGGGACACACCACTCTCAG | 93 |
| Pnpla2 |  | ACCACCCTTTCCAACATGCTA | GGCAGAGTATAGGGCACCA |  |
| Lipe |  | TGGCACACCATTTTGACCTG | TTGCGGTTAGAAGCCACATAG |  |
| Mgll |  | ACCATGCTGTGATGCTCTCTG | CAAACGCCTCGGGGATAACC |  |
| Plin1 |  | CGTGTAGTGTGGGGTCCTT | GGGCCCTTGTTCATTGACATC |  |
| Acaca |  | GATGAACCATCTCCGTTGGC | GACCCAATTATGAATCGGGAGTG |  |
| Fasn |  | GGAGGTGGTGATAGCCGGTAT | TGGGTAATCCATAGAGCCCAG |  |
| Scd1 |  | AGATCTCCAGTTCTTACACGACCAC | GACGGATGTCTTCTTCCAGGTG |  |
| Pdgfra |  | CAA ACC CTG AGA CCA CAA TG | TCC CCC AAC AGT AAC CCA AG |  |
| Pdgfrb |  | TGC CTC AGC CAA ATG TCA CC | TGC TCA CCA CCT CGT ATT CC |  |
| Zfp423 |  | GAGCCAGCACGCACAGTGAG | GCACACTAGCTGGAGCAGGAC |  |
| Dlk1 |  | CAGTGCATCTGCAAGGATGGCTG | CTTGTGCTGGCAGTCCTTTCCAG |  |
| Col1a1 |  | GTGCTCCTGGTATTGCTGGT | GGCTCCTCGTTTTCCTTCTT |  |
| Col3a1 |  | GGGTTTCCCTGGTCCTAAAG | CCTGGTTTCCCATTTTCTCC |  |
| Col6a3 |  | GATGAGGGTGAAGTGGGAGA | CAGCACGAAGAGGATGTCAA |  |
| Lox |  | CCACAGCATGGACGAATTCA | AGCTTGCTTTGTGGCCTTCA |  |
| Tlr4 |  | ATGGCATGGCTTACACCACC | GAGGCCAATTTTGTCTCCACA |  |
| Tnfa |  | CACGTCGTAGCAAACCACCAAGTGGA | TGGGAGTAGACAAGGTACAACCC |  |
| Adgre1 |  | CTTTGGCTATGGGCTTCCAGTC | GCAAGGAGGACAGAGTTTATCGTG |  |
| Il1b |  | CAACCAACAAGTGATATTCTCCATG | GATCCACACTCTCCAGCTGCA |  |
| Cd86 |  | TGTTTCCGTGGAGACGCAAG | TTGAGCCTTTGTAAATGGGCA |  |
| Cd163 |  | TCCACACGTCCAGAACAGTC | CCTTGGAAACAGAGACAGGC |  |
| Cd206 |  | CTCTGTTCAGCTATTGGACGC | CGGAATTTCTGGGATTCAGCTTC |  |
| Nfkb1 |  | ATG GCA GAC GAT GAT CCC TAC | TGT TGA CAG TGG TAT TTC TGG TG |  |
| Clec4e |  | CTT CGG GGA AGC AAC AAC TC | CAA GCA ACT GCA CCA GAG AAC |  |
| ß-Actin |  | GGC ACC ACA CCT TCT ACA ATG | GGG GTG TTG AAG GTC TCA AAC |  |
